# Phenotype-Based Stratification and Early Prediction of Staphylococcal Infective Endocarditis: Development of a Pragmatic Bedside Score

**DOI:** 10.3390/pathogens15040418

**Published:** 2026-04-13

**Authors:** Adina-Alexandra Nanu, Miruna-Ioana Lazăr, Dragoș Ștefan Lazăr, Corneliu Petru Popescu, Maria Nica, Simin Aysel Florescu

**Affiliations:** 1 Department of Infectious Diseases, “Carol Davila” University of Medicine and Pharmacy, 020021 Bucharest, Romania; adina.nanu@drd.umfcd.ro (A.-A.N.); dragos.lazar@umfcd.ro (D.Ș.L.); corneliu.popescu@umfcd.ro (C.P.P.); maria.nica@umfcd.ro (M.N.); simin.florescu@umfcd.ro (S.A.F.); 2 “Dr. Victor Babeș” Clinical Hospital of Infectious and Tropical Diseases, 030303 Bucharest, Romania; 3 “Nicolae Malaxa” Clinical Hospital, 022441 Bucharest, Romania

**Keywords:** IE, *Staphylococcus*, platelet kinetics

## Abstract

**Background**: Early identification of *Staphylococcus* spp. infective endocarditis (IE) remains clinically challenging but essential for timely initiation of targeted antimicrobial therapy. We aimed to characterize pathogen-specific clinical phenotypes and to develop a pragmatic bedside prediction model for staphylococcal etiology. **Methods**: We conducted a retrospective cohort study including 112 patients diagnosed with definite IE. Demographic, clinical, echocardiographic, and laboratory data were analyzed. Independent predictors of staphylococcal etiology were identified using multivariable logistic regression, with internal validation by bootstrap resampling. A simplified clinical risk score was derived from regression coefficients. Platelet kinetics were evaluated as a potential complementary biomarker. **Results**: Patients with staphylococcal IE (*n* = 66) were younger and exhibited a distinct clinical profile characterized by a high prevalence of intravenous drug use, right-sided valve involvement, and a markedly elevated inflammatory response. Independent predictors included intravenous drug use (OR 7.1, 95% CI 2.45–20.6, *p* < 0.001), higher C-reactive protein levels (OR 1.08 per unit increase, *p* = 0.025), and lower oxygen saturation (OR 0.75 per 1% increase, *p* = 0.007). The model demonstrated good discrimination (AUC 0.82, 95% CI 0.74–0.90) and calibration. The simplified exploratory risk score stratified patients into low-, intermediate-, and high-risk groups, with observed probabilities of 27%, 82%, and 94%, respectively. **Conclusions**: Staphylococcal IE represents a distinct clinical phenotype. A simple three-variable model enables early bedside identification of high-probability cases and may support risk-adapted management decisions. Nevertheless, external validation is required before clinical implementation of a score.

## 1. Introduction

Infective endocarditis (IE) remains a complex and heterogeneous disease in which the causative microorganism plays a pivotal role in determining clinical presentation, disease severity, and outcomes [1,2]. Contemporary epidemiological data identify *Staphylococcus* and *Streptococcus* species as the leading pathogens, with a growing predominance of staphylococcal infections in the context of healthcare exposure, invasive procedures, and intracardiac devices [2,3,4].

*Staphylococcus aureus*, in particular, is associated with an acute and aggressive disease course, characterized by rapid valvular destruction, intense systemic inflammation, and a high rate of complications, including embolic events and abscess formation [4,5,6]. In contrast, non-staphylococcal etiologies are more frequently associated with subacute presentations and underlying structural valve disease [4,7].

Despite these well-described differences, early etiological discrimination remains challenging in clinical practice, especially at initial presentation, when microbiological confirmation is not yet available [8,9]. Most existing studies have focused on outcomes or isolated predictors, while integrated analyses combining clinical, laboratory, and echocardiographic data remain limited [1,10]. Furthermore, few studies have translated such findings into simplified tools applicable at the bedside [2,8].

In this context, the aims of the present study were:

(1) to characterize pathogen-specific clinical phenotypes in IE,
(2) to identify independent predictors of staphylococcal etiology,
(3) to develop and internally validate a pragmatic clinical prediction model, and
(4) to explore platelet kinetics as a potential adjunctive biomarker.

## 2. Materials and Methods

### 2.1. Study Design and Population

This was a single-center retrospective cohort study including 112 consecutive adult patients diagnosed with definite IE according to the modified 2023 Duke criteria [11] and hospitalized between 2015 and 2025 in our tertiary care hospital in Bucharest, Romania. Patients hospitalized until May 2023 were reclassified to ensure homogenous cohort diagnostic criteria. Only patients fulfilling definite IE criteria and with positive cultures were finally included in the analysis.

Patients were classified into two groups based on immunological and microbiological findings: staphylococcal IE (isolation of *Staphylococcus* spp.) and non-staphylococcal IE (all other identified pathogens, such as *Streptococcus* spp., *Klebsiella* spp., *Coxiella burnetii*, *Bartonella henselae*, etc.).

### 2.2. Data Collection

Clinical and paraclinical data were extracted from electronic medical records. Variables included demographic characteristics, comorbidities, clinical and paraclinical findings and disease outcomes, as well as risk factors such as intravenous drug use (IVDU), tobacco use, and alcohol consumption. Comorbidity burden was calculated for each patient using the Charlson Comorbidity Index (CCI) [12]. Disease severity was assessed using the SOFA score [13,14]. Both were calculated online, using rapidly available tools like MDCalc version 5.4.9. (https://www.mdcalc.com).

Echocardiographic findings included valve involvement and vegetation characteristics (length, width). Laboratory parameters were recorded at admission (within 24 h) and at discharge for patients who survived. Regarding patients who died during hospitalization, the last set of laboratory results was included in the analysis. Platelet recovery was defined as the proportional change in platelet count during hospitalization and calculated as:

(discharge platelet count − admission platelet count)/admission platelet count.

### 2.3. Statistical Analysis

Statistical analysis was performed using R software version 4.4.1 and IBM SPSS Statistics v26, Chicago, IL, USA. Categorical variables are displayed as frequencies and percentages and were compared using the χ^2^ (chi-squared) test or Fisher’s exact test, as appropriate. Continuous variables are expressed as mean ± standard deviation or median (interquartile range), depending on normality of data distribution (assessed by Shapiro–Wilk and Kolmogorov–Smirnov tests), and were compared using Student’s *t*-test or Mann–Whitney *U* test, as appropriate. A *p*-value < 0.05 was considered statistically significant.

Independent predictors of staphylococcal etiology were identified using LASSO multivariable logistic regression. Model development followed current recommendations for prediction model studies (TRIPOD statement).

In order to evaluate model performance, we used discrimination (area under the ROC curve), calibration (calibration plots and mean absolute error), as well as clinical utility (decision curve analysis). Variable selection and model calibration were performed using LASSO regularization to reduce overfitting, considering an events-per-variable (EPV) ratio of 22 that exceeds the commonly recommended threshold of 10 events per variable. Internal validation was performed using bootstrap resampling (1000 iterations) to estimate model stability and optimism. A simplified clinical score was derived by transforming regression coefficients into integer values. Regression coefficients (β) from the final model were used as the basis for score construction.

Missing data were minimal (<2%) and were assumed to be missing at random. Given their limited frequency, a complete case analysis was undertaken. Sensitivity analyses confirmed that the exclusion of patients with incomplete data did not materially affect parameter estimates.

### 2.4. Ethical Considerations

In accordance with national regulations, all patients admitted to the hospital were informed about the potential use of their anonymized data for research purposes and had the opportunity to provide or decline consent at admission.

All data were handled in compliance with institutional ethical standards and applicable data protection regulations. The study protocol was approved by the local ethics committee of Victor Babes Clinical Hospital of Infectious and Tropical Diseases.

## 3. Results

### 3.1. Staphylococcal IE vs. Non-Staphylococcal IE

#### 3.1.1. Baseline Demographic and Risk Profile

Patients with *Staphylococcus* spp. IE displayed a distinct demographic and epidemiological profile compared with those with non-staphylococcal IE (Table 1). The staphylococcal group was significantly younger (48.8 ± 17.9 vs. 59.9 ± 16.2 years, *p* = 0.001), with a lower BMI (body mass index)—median 19.8 vs. 23.9 kg/m^2^, *p* = 0.003. No significant sex differences were observed between groups (*p* = 0.467). Additionally, the proportion of patients with a prior history of IE was similar (*p* = 0.758).

Behavioral risk factors were significantly more prevalent among patients with staphylococcal IE. Notably, intravenous drug use (IVDU) was markedly higher in the staphylococcal cohort (54.5% vs. 13%, *p* < 0.001), representing the most prominent differentiating factor between the two groups. Tobacco use was reported in 72.7% of cases compared to 41.3% in the non-staphylococcal group (*p* = 0.001), while alcohol consumption was also more frequent (62.1% vs. 32.6%, *p* = 0.002).

Consistent with this risk profile, blood-borne viral infections were significantly more common in patients with staphylococcal IE. HIV infection could be detected in 28.8% of cases in these patients compared to 10.9% in the non-staphylococcal group (*p* = 0.023), while hepatitis C infection showed an even stronger association (53% vs. 15.2%, *p* < 0.001). In contrast, no significant difference was observed in the prevalence of hepatitis B (*p* = 0.831).

Despite these marked epidemiological differences, the overall comorbidity burden, as assessed by the CCI, was not significantly different between groups (median 5 vs. 6, *p* = 0.983). Disease severity at presentation was significantly greater in the staphylococcal group, as reflected by the higher SOFA scores (median 2 vs. 1.5, *p* = 0.031), which indicate a more pronounced systemic impact at admission.

These baseline characteristics suggest that staphylococcal IE is associated with a younger, higher-risk population characterized by substance use and viral coinfections, alongside increased severity at initial presentation.

#### 3.1.2. Valvular Substrate and Echocardiographic Characteristics

Table 2 and Table 3 reveal that patients with non-staphylococcal IE were significantly more likely to have pre-existing valvular pathology, including stenosis or insufficiency (54.3% vs. 27.3%, *p* = 0.004), with aortic valve involvement being significantly more frequent in this group (39.1% vs. 18.2%, *p* = 0.014). In contrast, the prevalence of pre-existing prosthetic valves was not different between groups (16.7% vs. 17.4%, *p* = 0.920). These findings demonstrate an association between non-staphylococcal IE with structurally abnormal or previously damaged valves, consistent with the classical pathogenic paradigm of endothelial injury followed by microbial colonization. In contrast, staphylococcal IE appears less dependent on valvular abnormalities, supporting its recognized capacity to invade unaffected endocardium.

Clear differences in valve tropism were observed between groups, and echocardiographic evaluation (trans-thoracic or trans-esophageal, as needed) identified vegetations in the vast majority of cases, with no statistically significant difference in detection rates between groups (92.4% vs. 100%, *p* = 0.077), nor in the use of transthoracic or transesophageal echocardiography.

Tricuspid valve involvement was significantly more common in the staphylococcal group (43.9% vs. 17.4%, *p* = 0.003), whereas mitral valve infection predominated in non-staphylococcal IE (58.7% vs. 34.8%, *p* = 0.013). Aortic valve involvement was more prevalent in the non-staphylococcal group, although not reaching statistical significance (24.2% vs. 39.1%, *p* = 0.092). The affection of the pulmonary valve was rather rare in both groups.

Infection occurring on previously damaged valves was significantly more frequent in non-staphylococcal IE (45.7% vs. 22.7%, *p* = 0.011), further supporting the role of structural predisposition in this subgroup. The association between a history of valve impairment and endocarditis affecting insufficient or stenotic valves is significant (*p* < 0.001) within each group.

Vegetation size demonstrated a trend toward larger dimensions in the staphylococcal group, particularly in the longitudinal axis (median 13 vs. 10 mm), a difference that, nevertheless, did not reach the significance threshold (*p* = 0.056). No differences were observed in transverse diameter or in the incidence of multiple valve involvement.

These findings highlight distinct pathogen-specific patterns of valvular involvement, with staphylococcal IE preferentially affecting the right endocardium and structurally normal valves, and non-staphylococcal IE more commonly associated with left-sided disease on pre-existing valvular lesions.

#### 3.1.3. Clinical Presentation

Patients with staphylococcal IE presented with more evident systemic inflammatory syndrome signs compared to those with non-staphylococcal IE (Table 4). Although peak body temperature values did not differ significantly between groups (*p* = 0.225), fever was significantly more common in the staphylococcal group (90.9% vs. 67.4%, *p* = 0.002).

Respiratory involvement through symptoms like cough (50% vs. 21.7%, *p* = 0.002) and abnormal lung auscultation findings (50% vs. 17.4%, *p* < 0.001) was more evident in staphylococcal IE. In addition, this group exhibited significantly lower peripheral oxygen saturation at admission (median 96% vs. 98%, *p* < 0.001), suggesting a higher burden of pulmonary involvement. Hepatomegaly as another sign of systemic involvement had a higher incidence in these patients (47% vs. 19.6%, *p* = 0.003), while other clinical features such as dyspnea, peripheral edema, chest pain, fatigue, syncope, and hypotension did not differ significantly between groups. Cardiac systolic murmurs were also similarly distributed (*p* = 0.143).

This clinical profile suggests a more aggressive, systemically disseminated presentation in staphylococcal IE, likely reflecting the higher prevalence of right-sided involvement and septic pulmonary embolization.

#### 3.1.4. Laboratory Markers of Inflammation and Organ Dysfunction

At hospital admission, patients with staphylococcal positive cultures demonstrated a significantly more intense inflammatory and tissue injury profile (Table 5), reflected by higher levels of CRP (C-reactive protein—*p* = 0.001), procalcitonin (*p* = 0.009), D-dimers (*p* = 0.015), and lactate dehydrogenase (*p* < 0.001). Markers of hepatic involvement were also significantly more elevated, including aspartate aminotransferase (*p* = 0.001) and total bilirubin (*p* = 0.021).

Thrombocytopenia was significantly more pronounced in the staphylococcal group (*p* < 0.001), consistent with a more severe inflammatory and pro-thrombotic state. No statistically significant differences were observed in leukocyte count, fibrinogen, hemoglobin, creatinine, or troponin levels at admission.

At discharge (Table 6), patients with staphylococcal IE continued to exhibit elevated D-dimers (*p* = 0.006) and increased lactate dehydrogenase levels (*p* = 0.004), along with higher leukocyte counts (*p* = 0.017), suggesting ongoing inflammatory and metabolic activity despite treatment. In contrast, fibrinogen levels were significantly higher in the non-staphylococcal group at discharge (*p* = 0.011), potentially reflecting a more prolonged, subacute inflammatory response.

Other laboratory parameters did not differ significantly between groups at discharge.

#### 3.1.5. Complications and Clinical Outcomes

Embolic complications arose as the most notable difference in clinical course between the two groups (Table 7), with significantly more embolic events in the staphylococcal IE group compared to non-staphylococcal IE (54.5% vs. 30.4%, *p* = 0.012). This result is consistent with the recognized higher embolic potential of staphylococcal vegetations, likely related to their rapid growth, friable structure, and increased propensity for tissue destruction. However, specific organ affection by systemic emboli did not reach statistical significance between groups, with the exception of pulmonary embolism, associated with Staphylococcal IE (*p* = 0.001).

Despite a higher burden of embolic complications in the staphylococcal cohort, rates of intensive care unit admission (25.8% vs. 13%, *p* = 0.101), need for cardiac surgery consultation (21.2% vs. 21.7%, *p* = 0.947), and surgical intervention (10.6% vs. 17.4%, *p* = 0.300) did not differ significantly between groups. Similarly, the length of hospital stay was comparable (median 31.5 vs. 30 days, *p* = 0.568), as well as discharge outcomes. In-hospital mortality was higher in the staphylococcal group (18.2% vs. 8.7%), although this difference did not reach statistical significance (*p* = 0.158).

These findings suggest that while staphylococcal IE is associated with a significantly increased risk of embolic complications, this did not translate into statistically significant differences in short-term in-hospital outcomes within the present cohort.

### 3.2. Predictors of Staphylococcal Etiology

#### 3.2.1. Multivariable Logistic Regression Model

A multivariable logistic regression model (Table 8) was constructed to identify independent predictors of *Staphylococcus* spp. IE. Three variables remained significantly associated with staphylococcal etiology: intravenous drug use (IVDU), C-reactive protein (CRP) level at admission, and peripheral oxygen saturation (SaO_2_). IVDU emerged as the strongest independent predictor, being associated with a more than seven-fold increase in the odds of staphylococcal IE (OR 7.10, 95% CI 2.45–20.61, *p* < 0.001).

Higher CRP levels were also independently associated with staphylococcal infection (OR 1.08 per unit increase, 95% CI 1.01–1.15, *p* = 0.025), reflecting the more pronounced inflammatory response observed in this group.

In addition, staphylococcal infection was significantly associated with lower SaO_2_ (%) at presentation, each 1% increase being associated with a 25% reduction in odds (OR 0.75, 95% CI 0.60–0.92, *p* = 0.007).

**Figure 1:**
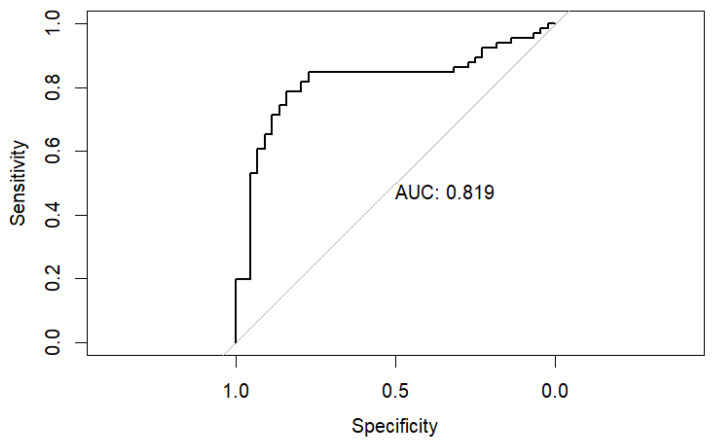
ROC analysis of multivariable logistic regression model for prediction of staphylococcal etiology of IE. Black stepped line: ROC curve of the multivariable logistic regression model for predicting *Staphylococcus* IE (independent predictors: IVDU, admission CRP level, and SaO_2_. Gray diagonal line: Reference line of no discrimination (random classifier, AUC = 0.5). Model performance—AUC = 0.819 (95% CI 0.74–0.90; DeLong test *p* < 0.001 vs. chance). Optimal probability threshold = 0.55 → sensitivity 78.8%, specificity 84.1%. The model was internally validated with 1000 bootstrap resamples (all predictors remained significant: IVDU *p* = 0.002; CRP *p* = 0.017; SaO_2_ *p* = 0.004). IVDU was the strongest predictor (OR 7.10, 95% CI 2.45–20.61), followed by higher CRP (OR 1.08 per unit) and lower SaO_2_ (OR 0.75 per 1% increase). Brier score = 0.1585674.

The robustness of these findings was confirmed using bootstrap validation with 1000 resamples. All three variables remained statistically significant predictors, with consistent effect sizes (intravenous drug use: *p* = 0.002; CRP: *p* = 0.017; peripheral oxygen saturation: *p* = 0.004), supporting the internal stability of the model. Model discrimination was evaluated using receiver operating characteristic (ROC) analysis, demonstrating good performance with an area under the curve (AUC) of 0.82 (95% CI 0.74–0.90)—Figure 1. This was significantly superior to chance prediction (DeLong test Z = 7.47, *p* < 0.001). The optimal probability threshold of 0.55 yielded a sensitivity of 78.8% and specificity of 84.1%.

The performance of the logistic regression models was assessed using LASSO logistic regression, which yielded an AUC of 0.820 (95% CI: 0.736–0.903). DeLong’s test showed no statistically significant difference between the two AUCs (Z = −0.181, *p* = 0.856), indicating that LASSO maintained the discriminatory performance of the original model.

**Figure 2:**
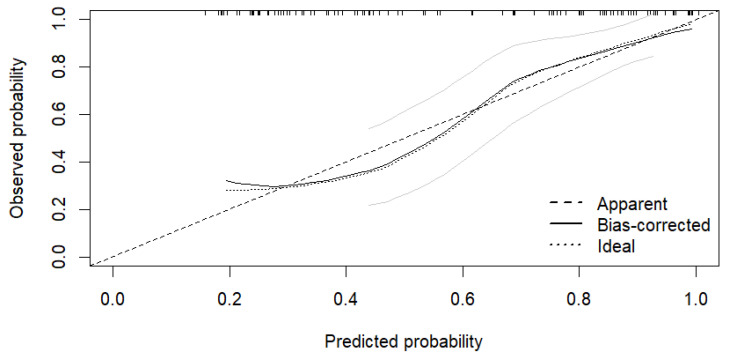
Calibration Curve of the Multivariable Prediction Model for *Staphylococcus* spp. IE. The apparent (dotted line), bias-corrected (solid line), and ideal (dashed line) curves are shown based on 1000 bootstrap resamples. Grey lines represent bootstrap-derived uncertainty bands and reflect reasonably stable calibration. Mean absolute calibration error = 0.038; 90th percentile = 0.074. Brier score = 0.159. The Brier score indicates good overall predictive accuracy of the model.

Model calibration showed good agreement between predicted and observed probabilities. The mean absolute calibration error was 0.038, indicating that predicted risks deviated from observed outcomes by an average of 3.8%. Furthermore, the 90th percentile of absolute error was 0.074, supporting stable calibration across the full range of predicted probabilities. Bootstrap-based calibration analysis (1000 resamples) confirmed good concordance between apparent and bias-corrected estimates (Figure 2). The Brier score was 0.159, indicating good overall predictive accuracy.

Although the Hosmer–Lemeshow test suggested statistical lack of fit (*p* < 0.05), this result should be interpreted with caution, given the known limitations of this test in small samples and well-performing models.

#### 3.2.2. Decision Curve Analysis of Multivariable Logistic Regression Model

Decision curve analysis (DCA) demonstrated that the multivariable prediction model provided a greater net clinical benefit compared to both treat-all and treat-none strategies across a wide range of threshold probabilities (approximately 0.16–0.99).

**Figure 3:**
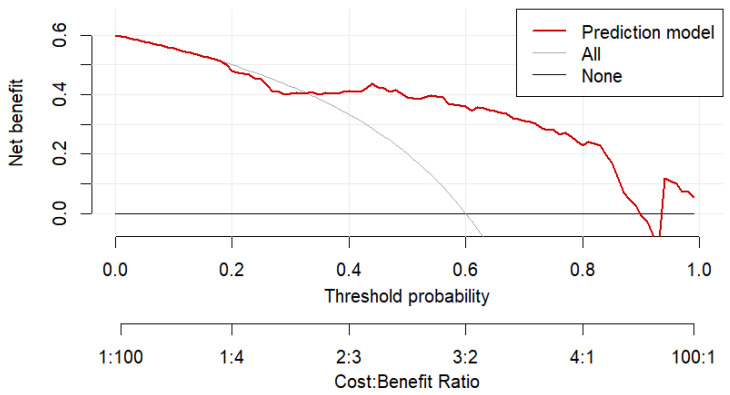
DCA: Net Clinical Benefit of the Multivariable Prediction Model for staphylococcal IE Compared with Treat-All and Treat-None Strategies. Red line: Net benefit curve of the multivariable logistic regression model for predicting *Staphylococcus* spp. IE (independent predictors: IVDU, admission CRP, and SaO_2_). Gray line: Net benefit of the “treat-all” strategy. Black line: Net benefit of the “treat-none” strategy.

**Figure 4:**
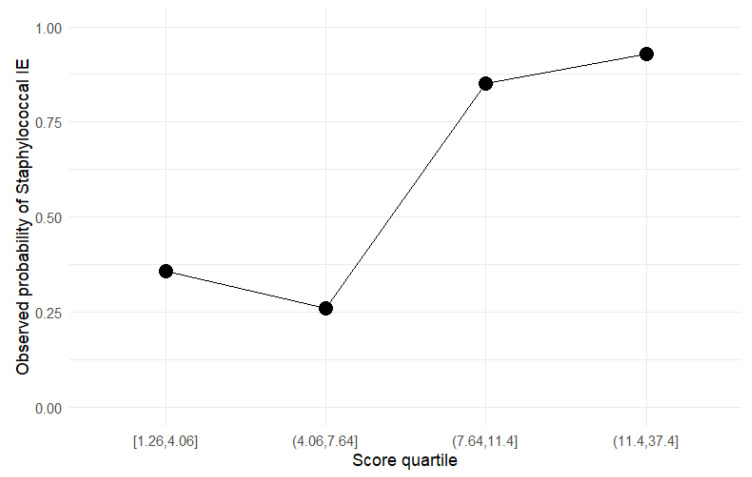
Observed event rates of *Staphylococcus* etiology are presented across quartiles of predicted risk derived from the multivariable model (IVDU, admission CRP, and SaO_2_%). Points represent observed proportions within each quartile. The model demonstrates appropriate calibration, with increasing observed probabilities across ascending risk categories, supporting its ability to stratify patients according to the likelihood of staphylococcal infection.

These findings indicate that the model has potential clinical utility in supporting early identification of patients with staphylococcal IE and may aid in risk-informed decision-making (Figure 3 and Figure 4).

#### 3.2.3. Clinical Risk Score

Using the multivariable logistic regression model, a bedside clinical score was derived by transforming regression coefficients into integer-based weights and thus preserving the relative contribution of each independent predictor. Continuous variables were scaled to clinically meaningful units (CRP per 10 mg/dL and SaO_2_ per 1% decrease from 100%) to improve interpretability. The resulting point allocation closely approximates the ratio of regression coefficients (approximately 7:2.5:1), with minor simplification to improve clinical usability. For each patient, 5 points are assigned for a history of intravenous drug use, 2 points are added for every 10 mg/dL increase in CRP, and 1 point is added for each percentage decrease in oxygen saturation below 100%. The total score corresponds to a probability of staphylococcal infective endocarditis, allowing rapid risk stratification before microbiological confirmation (Table 9).

The score can be calculated at admission using routinely available clinical and paraclinical data and is derived from this formula:

Staphylococcal Prediction Score = 5 × (IVDU) + 2 × (CRP/10) + (100 − SaO_2_).

**Figure 5:**
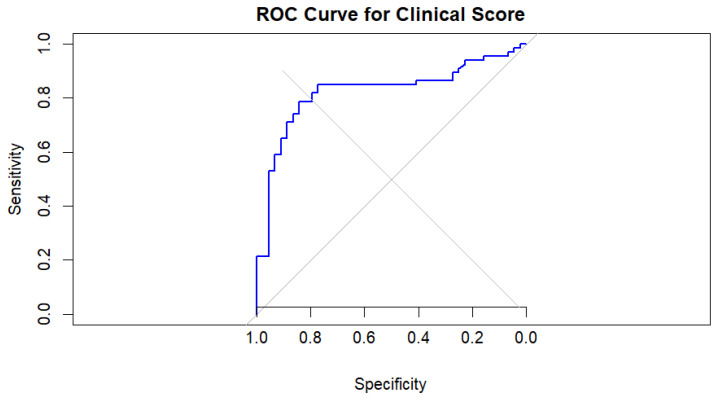
Receiver Operating Characteristic (ROC) Curve for the Staphylococcal Prediction by multivariable logistic regression model. Stepped blue line: model’s discriminative performance; grey diagonal line: chance-level discrimination (AUC). AUC = 0.8228, 95% CI: 0.7404–0.9053 (DeLong). Optimal threshold—6.93 (Youden’s Index), Specificity = 84.09%, Sensitivity = 78.8% (Youden method). Brier score = 0.1577727.

Stratification according to the derived clinical score identified three clearly distinct risk categories, with a progressive increase in the probability of *Staphylococcus* spp. IE across groups. The clinical score demonstrated excellent discrimination (AUC of 0.8228 (95% CI 0.7404–0.9053—DeLong) and robust risk stratification (Figure 5). The lower threshold of 7 points closely matched the optimal Youden-derived cut-off (6.93), supporting its data-driven validity. A score ≥7 provided balanced sensitivity (78.8%) and specificity (84.1%), while a higher threshold (>11) identified a subgroup with very high specificity (95.5%), consistent with a rule-in strategy. Importantly, score thresholds resulted in complete separation between risk groups without overlap. Observed probabilities increased sharply above 7 points and plateaued at higher values, confirming the clinical relevance of the selected categories.

Patients in the low-risk category had an observed probability of 27% for staphylococcal etiology, suggesting that *Staphylococcus* infection is less likely in this subgroup. The intermediate-risk group demonstrated a substantially higher probability of 82%, indicating a strong clinical suspicion. Patients classified as high-risk exhibited a 94% probability of staphylococcal IE, reflecting a very high likelihood of this etiology (Table 10).

Overall, this stepwise gradient in risk supports the clinical relevance of the score for patient stratification and may facilitate early, targeted diagnostic and therapeutic decision-making.

A DeLong test for correlated receiver operating characteristic (ROC) curves showed no statistically significant difference between the full multivariable model and the simplified clinical score (*Z* = 0.75, *p* = 0.456), confirming that the simplified score preserves the discriminatory performance of the original model without meaningful loss of information.

### 3.3. Platelet Dynamics During Hospitalization as a Complementary Biomarker

Significant differences in platelet dynamics were observed between patients with staphylococcal and non-staphylococcal IE (Table 11). Patients with staphylococcal IE demonstrated a significantly greater increase in platelet counts during hospitalization compared to those with non-staphylococcal etiology. The median absolute change in platelet count (Δ platelets) was +43,000/µL (IQR 143,250) in the staphylococcal group, whereas a decline was observed in the non-staphylococcal group (−35,000/µL, IQR 141,000), with this difference reaching statistical significance threshold (*U* = 1037.5, *p* = 0.007).

Similarly, platelet recovery—defined as the proportional change in platelet count between admission and discharge—was significantly higher in patients with staphylococcal IE. The median platelet recovery was 0.246 (IQR 1.57) in the staphylococcal group, compared to −0.102 (IQR 0.562) in the non-staphylococcal group (*U* = 1000.5, *p* = 0.004).

**Figure 6:**
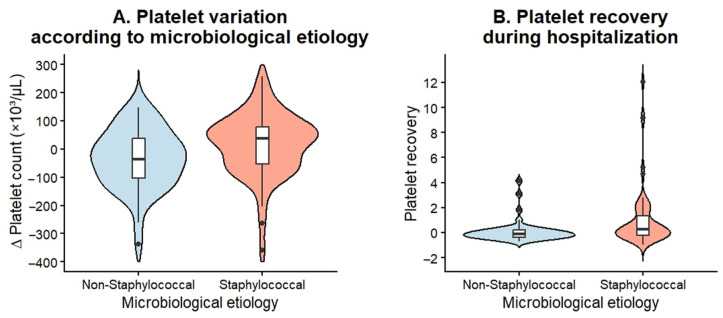
Violin plots illustrating the distribution of platelet count variation (Δ platelet count) and platelet recovery during hospitalization among patients with IE caused by *Staphylococcus* and non-staphylococcal pathogens. Panel (**A**) shows the absolute change in platelet count between admission and hospital discharge expressed as Δ platelet count (×10^3^/µL). Panel (**B**) presents platelet recovery, defined as the proportional change in platelet count during hospitalization, calculated as (platelet count at discharge − platelet count at admission)/platelet count at admission.

These findings were further supported by distribution analyses (Figure 6), which demonstrated a clear shift toward platelet recovery in staphylococcal IE, in contrast to a trend toward platelet decline in non-staphylococcal cases. These results further highlight distinct platelet kinetics associated with staphylococcal IE, suggesting a differential hematological response during hospitalization that may reflect underlying differences in inflammatory and thrombotic pathways.

**Figure 7:**
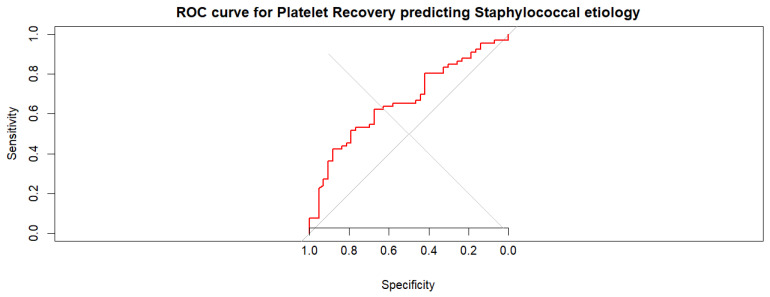
Receiver operating characteristic (ROC) curve evaluating the ability of platelet recovery to discriminate staphylococcal IE from non-staphylococcal etiologies. Platelet recovery demonstrated moderate discriminatory performance with an AUC of 0.663 (95% CI 0.562–0.765). Optimal cut-off value—0.22 (determined by the Youden index), yielding 51.5% sensitivity and 80.0% specificity for identifying staphylococcal IE. Grey diagonal line: chance-level discrimination (AUC).

**Figure 8:**
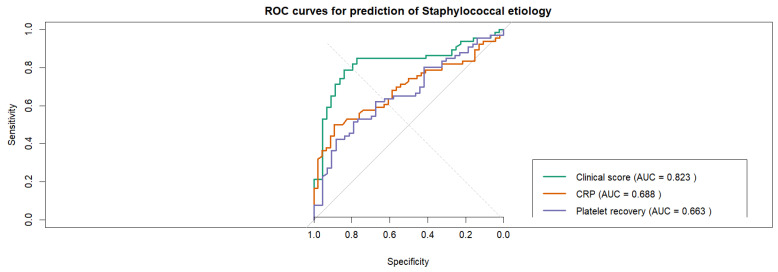
Comparison of ROC curves for predictors of *Staphylococcus* etiology in IE. The clinical prediction score demonstrated the highest discriminative performance, followed by C-reactive protein (CRP) at admission and platelet recovery. Platelet recovery showed moderate discriminatory ability, indicating that platelet kinetics carry pathogen-specific information but are less accurate than multivariable clinical prediction models. Grey diagonal line: chance-level discrimination (AUC).

ROC analysis demonstrated that platelet recovery had moderate discriminatory ability for identifying IE caused by *Staphylococcus* spp., with an AUC of 0.663 (95% CI 0.562–0.765)—Figure 7. In contrast, the derived clinical prediction score demonstrated superior discriminatory performance, with an AUC of 0.823. Direct comparison of ROC curves using DeLong’s test confirmed that the prediction model performed significantly better than platelet recovery alone (AUC 0.823 vs. 0.663, *p* = 0.017)—Figure 8.

## 4. Discussion

In this study, we identified two distinct clinical phenotypes of IE according to microbiological etiology, with *Staphylococcus* spp. infection characterized by a younger demographic profile, a more intense inflammatory response, and a higher burden of embolic complications compared with non-staphylococcal IE [1,2]. In addition, we developed and internally validated a multivariable prediction model based on readily available admission variables, which demonstrated good discrimination, calibration, and potential clinical utility for early identification of staphylococcal etiology.

A key finding of our study is that early etiological discrimination can be achieved using a limited set of variables that reflect acute pathophysiological processes rather than baseline comorbidities. Intravenous drug use, systemic inflammatory burden (as reflected by CRP), and impaired oxygenation (SaO_2_) captured most of the predictive signal for staphylococcal IE, consistent with prior observations linking these factors to staphylococcal bacteremia and severe infection [15,16,17,18,19,20]. These variables likely integrate both exposure risk and downstream organ involvement, suggesting that early clinical phenotype may provide more actionable information than traditional comorbidity-based approaches.

Consistent with established pathophysiological paradigms, we observed marked differences in valvular involvement. Non-staphylococcal IE was more frequently associated with pre-existing valvular disease and aortic valve involvement, whereas staphylococcal infection showed a clear predilection for the tricuspid valve and often occurred in structurally normal valves. This pattern likely reflects both the virulence of *Staphylococcus* spp. and the high prevalence of IVDU, with direct inoculation into the venous circulation leading to right-sided disease.

From a clinical perspective, staphylococcal IE was associated with a more pronounced systemic inflammatory phenotype, including higher rates of fever, respiratory symptoms, abnormal pulmonary findings, hepatomegaly, and lower oxygen saturation. These findings were paralleled by significantly elevated inflammatory and tissue injury markers at admission, including CRP, procalcitonin, D-dimers, and LDH, as well as more pronounced thrombocytopenia [15,16,17,21]. The persistence of several abnormalities at discharge suggests a more sustained inflammatory response in this group.

The higher incidence of embolic complications in the staphylococcal group is consistent with prior literature and likely reflects the biological characteristics of staphylococcal vegetations. However, our findings suggest that this association is not solely pathogen-driven. Rather, embolic risk appears to be mediated by a combination of host-related factors, including IVDU, inflammatory burden, and valve involvement, supporting the concept that embolization in IE is a multifactorial process [15,17,18,19,20,22].

An important contribution of this study is the development of a parsimonious multivariable model incorporating three routinely available variables. IVDU emerged as the strongest predictor, consistent with its well-established association with staphylococcal bacteremia. Notably, peripheral oxygen saturation—although not traditionally included in prediction models—likely reflects the presence of pulmonary septic emboli and right-sided involvement, providing a physiologically meaningful marker of disease expression. The model demonstrated good discrimination (AUC ≈ 0.82) and satisfactory calibration, supporting its potential clinical applicability in the early phase of evaluation. Subgroup analysis excluding patients with IVDU demonstrated that CRP and oxygen saturation retained moderate discriminatory performance (AUC 0.723, *p* = 0.002), suggesting that the model is not solely driven by drug use status.

To facilitate bedside use, we derived a simplified clinical score based on the regression coefficients. While this score allowed stratification into clinically meaningful risk categories, it should be regarded as a pragmatic approximation of the full model rather than an independently validated tool. These findings require validation in external cohorts before clinical implementation. The score was not independently validated and should be considered exploratory.

In addition to clinical predictors, our study explored platelet kinetics as a potential complementary biomarker. Patients with staphylococcal IE demonstrated significantly greater platelet recovery during hospitalization compared with non-staphylococcal cases. This pattern likely reflects pathogen-specific interactions between bacteria and platelets, as *Staphylococcus aureus* is known to promote platelet activation, aggregation, and consumption within vegetations and microthrombi, followed by recovery with effective antimicrobial therapy [15,16,23,24,25]. Platelet recovery showed only moderate discriminatory ability (AUC ≈ 0.66), its relatively high specificity suggesting a potential supportive role when interpreted alongside clinical variables. Platelet recovery should be interpreted as a dynamic biomarker reflecting pathogen-specific host–platelet interactions rather than a standalone diagnostic discriminator. Its moderate performance and delayed availability limit its utility in early etiological prediction; however, it may provide complementary insight into disease evolution and treatment response.

These findings support a phenotype-oriented view of IE, in which microbiological etiology reflects the interaction between host exposure, pathogen virulence, and organ-level involvement. The “staphylococcal phenotype” identified in our cohort—characterized by behavioral risk factors, right-sided disease, systemic inflammation, and pulmonary compromise—may be clinically recognizable prior to microbiological confirmation, with potential implications for early therapeutic decision-making.

From a clinical standpoint, early identification of staphylococcal etiology may influence empirical antimicrobial selection, monitoring intensity, and diagnostic suspicion for complications such as septic pulmonary embolism. A simple model based on readily available variables may therefore support decision-making during the initial phase of care, when microbiological data are not yet available.

Several limitations should be acknowledged. The retrospective, single-center design may limit generalizability, and the modest sample size increases the risk of overfitting and limits statistical power for secondary outcomes. However, we addressed potential overfitting by applying LASSO regularization and performing internal validation using bootstrap resampling. Moreover, the EPV ratio exceeded the recommended threshold of 10 events per variable. Residual confounding cannot be excluded, and external validation is necessary to confirm robustness and clinical implementation feasibility.

In addition, the non-staphylococcal IE group was microbiologically heterogeneous, including primarily *Streptococcus* spp. and a few Gram-negative organisms, with no cases of HACEK IE. Grouping these diverse pathogens together as a single comparator may have obscured pathogen-specific differences and could have influenced model performance. A detailed breakdown of the non-staphylococcal organisms is provided in the Supplementary Materials.

## 5. Conclusions

In conclusion, staphylococcal IE can be recognized early as a distinct clinical phenotype characterized by specific epidemiological, physiological, and inflammatory features. A simple model incorporating IVDU, CRP, and oxygen saturation provides robust and clinically applicable discrimination of staphylococcal etiology prior to microbiological confirmation, although external validation is required before clinical implementation.

These findings support a phenotype-based approach to early decision-making in infective endocarditis and highlight the potential of integrating dynamic biomarkers, such as platelet kinetics, into future predictive frameworks.

## Figures and Tables

**Table 1:** Demographic and baseline characteristics of Staphylococcal and Non-staphylococcal groups.

	*Staphylococcus * spp. IE (*n* = 66)	Non-*Staphylococcus* spp. IE (*n* = 46)	Test Statistic Value	*p*-Value
Females, *n* (%)	16 (24.2%)	14 (30.4%)	*ꭓ*^2^ = 0.530	0.467 ^a^
Males, *n* (%)	50 (75.8%)	32 (69.6%)		
Age (years) mean ± SD	48.7 ± 17.9	59.9 ± 16.2	*t* = 3.358	0.001
BMI—kg/m^2^, median (IQR)	19.8 (18.3–25.6)	23.8 (20.6–29)	*U* = 708, *Z* = −2.986	0.003
History of IE, *n* (%)	6 (9.1%)	5 (10.9%)	-	0.758 ^b^
Past or active tobacco use, *n* (%)	48 (72.7%)	19 (41.3%)	*ꭓ*^2^ = 11.136	0.001 ^a^
Alcohol consumption, *n* (%)	41 (62.1%)	15 (32.6%)	*ꭓ*^2^ = 9.444	0.002 ^a^
IVDU, *n* (%)	36 (54.5%)	6 (13.0%)	*ꭓ*^2^ = 19.921	<0.001 ^a^
CCI score, median (IQR)	5 (2–7)	6 (1–7)	*U* = 1521, *Z* = 0.021	0.983
SOFA score, median (IQR)	2 (1–4)	1.5 (0–3)	*U* = 1876.5, *Z* = 2.158	0.031
HIV infection, *n* (%)	19 (28.8%)	5 (10.9%)	*ꭓ*^2^ = 5.169	0.023 ^a^
Hepatitis B infection, *n* (%)	5 (7.6%)	3 (6.5%)	*ꭓ*^2^ = 0.045	0.831 ^a^
Hepatitis C infection, *n* (%)	35 (53.0%)	7 (15.2%)	*ꭓ*^2^ = 16.537	<0.001 ^a^
*Staphylococcus aureus* IE, *n* (%)	55 (83.3%)	-	-	-
CoNS, *n* (%)	11 (16.6%)	-	-	-

^a^—Chi-squared test, ^b^—Fisher’s exact test, IVDU—intravenous drug use; CoNS—coagulase-negative staphylococci.

**Table 2:** Valvular characteristics between groups.

	*Staphylococcus* spp. IE (*n* = 66)	Non-*Staphylococcus* spp. IE (*n* = 46)	Test Statistic Value	*p*-Value
Prosthetic valve, *n* (%)	11 (16.7%)	8 (17.4%)	*ꭓ*^2^ = 0.010	0.920 ^a^
Prior valve stenosis or insufficiency, *n* (%)	18 (27.3%)	25 (54.3%)	*ꭓ*^2^ = 8.401	0.004 ^a^
MV, *n* (%)	12 (18.2%)	15 (32.6%)	*ꭓ*^2^ = 3.084	0.079 ^a^
TV, *n* (%)	3 (4.5%)	3 (6.5%)	*ꭓ*^2^ = 0.209	0.688 ^a^
AV, *n* (%)	12 (18.2%)	18 (39.1%)	*ꭓ*^2^ = 6.066	0.014 ^a^
PV, *n* (%)	1 (1.5%)	1 (2.2%)	-	0.067 ^b^

^a^—Chi-squared test, ^b^—Fisher’s exact test.

**Table 3:** Echocardiographic characteristics of valve vegetations—comparison between groups.

	*Staphylococcus* spp. IE (*n* = 66)	Non-*Staphylococcus* spp. IE (*n* = 46)	Test Statistic Value	*p*-Value
Identified vegetation, *n* (%)	61 (92.4%)	46 (100.0%)	-	0.077 ^b^
TTE, *n* (%)	49 (74.2%)	34 (73.9%)	*ꭓ*^2^ = 0.002	0.969 ^a^
TEE, *n* (%)	17 (25.8%)	15 (32.6%)	*ꭓ*^2^ = 0.634	0.430 ^a^
Longitudinal size—mm, median (IQR)	13.0 (8.0–18.5)	10.0 (7.0–15.0)	*U* = 895, *Z* = 1.910	0.056
Transverse width—mm, median (IQR)	10.0 (5.0–14.5)	8.0 (5.0–11.5)	*U* = 838, *Z* = 1.330	0.184
Multiple valve involvement, *n* (%)	2 (3.0%)	2 (4.3%)	-	1.000 ^b^
IE of MV, *n* (%)	23 (34.8%)	27 (58.7%)	*ꭓ*^2^ = 6.238	0.013 ^a^
IE of TV, *n* (%)	29 (43.9%)	8 (17.4%)	*ꭓ*^2^ = 8.636	0.003 ^a^
IE of AV, *n* (%)	16 (24.2%)	18 (39.1%)	*ꭓ*^2^ = 2.842	0.092 ^a^
IE of PV, *n* (%)	0 (0%)	0 (0%)	-	-
Prosthetic valve IE, *n* (%)	11 (16.7%)	8 (17.4%)	*ꭓ*^2^ = 0.010	0.920 ^a^
IE affecting valves with prior stenosis or insufficiency, *n* (%)	15 (22.7%)	21 (45.7%)	*ꭓ*^2^ = 6.532	0.011 ^a^

^a^—Chi-squared test, ^b^—Fisher’s exact test. TEE—transesophageal echography; TTE—transthoracic echography.

**Table 4:** Clinical presentation and signs—comparison between the two groups.

	*Staphylococcus* spp. IE (*n* = 66)	Non-*Staphylococcus* spp. IE (*n* = 46)	Test Statistic Value	*p*-Value
Fever, *n* (%)	60 (90.9%)	31 (67.4%)	*ꭓ*^2^ = 9.841	0.002 ^a^
Maximum body temperature—°C, median (IQR)	38.6 (38.0–39.2)	39.2 (37.4–39.2)	*U* = 1355, *Z* = 1.213	0.225
Dyspnea, *n* (%)	25 (37.9%)	11 (23.9%)	*ꭓ*^2^ = 2.424	0.119 ^a^
Cough, *n* (%)	33 (50.0%)	10 (21.7%)	*ꭓ*^2^ = 9.153	0.002 ^a^
Peripheral edema, *n* (%)	13 (19.7%)	10 (21.7%)	*ꭓ*^2^ = 0.069	0.792 ^a^
Chest pain, *n* (%)	7 (10.6%)	5 (10.9%)	-	1.000 ^b^
Fatigue, *n* (%)	61 (92.4%)	44 (95.7%)	-	0.698 ^b^
Syncope, *n* (%)	5 (7.6%)	4 (8.7%)	-	0.830 ^b^
Systolic AP < 90 mmHg, *n* (%)	6 (9.1%)	4 (8.7%)	-	1.000 ^b^
RR, median (IQR)	20 (17–25)	18 (17–20)	*U* = 1557, *Z* = 1.621	0.105
SaO_2_%, median (IQR)	96.00 (93.00–97.25)	98.00 (96.00–98.00)	*U* = 848.5, *Z* = −3.747	<0.001
Lung sounds, *n* (%)	33 (50.0%)	8 (17.4%)	*ꭓ*^2^ = 12.421	<0.001 ^a^
Murmurs, *n* (%)	40 (60.6%)	34 (73.9%)	*ꭓ*^2^ = 2.141	0.143 ^a^
Lymphadenopathy, *n* (%)	20 (30.3%)	7 (15.2%)	*ꭓ*^2^ = 3.372	0.066 ^a^
Enlarged liver, *n* (%)	31 (47.0%)	9 (19.6%)	*ꭓ*^2^ = 8.867	0.003 ^a^

^a^—Chi-squared test, ^b^—Fisher’s exact test. RR—respiratory rate.

**Table 5:** Laboratory parameters at admission—comparison between the two groups.

	*Staphylococcus* spp. IE (*n* = 66)	Non-*Staphylococcus* spp. IE (*n* = 46)	Test Statistic Value	*p*-Value
Leukocytes × 10^3^/µL, median (IQR)	12.70 (8.65–17.07)	10.80 (7.67–13.10)	*U* = 1814, *Z* = 1.751	0.080
CRP—mg/dL, median (IQR)	12.85 (5.73–19.52)	6.71 (4.17–11.07)	*U* = 2087.5, *Z* = 3.368	0.001
Procalcitonin—ng/mL, median (IQR)	1.50 (0.23–5.20)	0.25 (0.12–0.90)	*U* = 1411.5, *Z* = 2.619	0.009
Fibrinogen—mg/dL, median (IQR)	513.50 (361.75–652.00)	534.00 (469.50–641.50)	*U* = 1299.5, *Z* = −1.114	0.265
Hemoglobin—g/dL, median (IQR)	9.70 (8.77–11.02)	10.40 (9.47–11.32)	*U* = 1201.5, *Z* = −1.873	0.061
Thrombocytes × 10^3^/µL, median (IQR)	166.50 (74.76–263.65)	260 (205.50–326.25)	*U* = 908, *Z* = −3.608	<0.001
Ultrasensitive Troponin I—ng/mL, median (IQR)	48.40 (23.05–111.5)	29.45 (6.07–56.17)	*U* = 625, *Z* = −1.786	0.074
D-dimers—ng/mL, median (IQR)	3.50 (1.82–5.31)	1.83 (1.01–3.65)	*U* = 824.5, *Z* = 2.423	0.015
Creatinine—mg/dL, median (IQR)	1.00 (0.72–1.41)	1.09 (0.76–1.39)	*U* = 1492, *Z* = −0.154	0.878
GOT—U/L, median (IQR)	44.00 (26.00–66.00)	26.50 (17.25–48.75)	*U* = 1947.5, *Z* = 3.197	0.001
LDH—U/L, median (IQR)	310.00 (240.25–460.75)	226.50 (185.75–279.50)	*U* = 1486.5, *Z* = 3.671	<0.001
Total bilirubin—mg/dL, median (IQR)	0.88 (0.51–1.32)	0.54 (0.35–0.95)	*U* = 1299.5, *Z* = 2.317	0.021

GOT—glutamic oxaloacetic transaminase, LDH—lactate dehydrogenase.

**Table 6:** Laboratory parameters at discharge—comparison between the two groups.

	*Staphylococcus* spp. IE (*n* = 66)	Non-*Staphylococcus* spp. IE (*n* = 46)	Test Statistic Value	*p*-Value
Leukocytes × 10^3^/µL, median (IQR)	9.50 (6.72–13.07)	7.70 (5.50–11.50)	*U* = 1881, *Z* = 2.379	0.017
CRP—mg/dL, median (IQR)	2.01 (0.54–8.87)	1.75 (0.57–7.32)	*U* = 1567, *Z* = 0.493	0.622
Procalcitonin—ng/mL, median (IQR)	0.15 (0.05–0.52)	0.10 (0.02–0.37)	*U* = 929, *Z* = 1.082	0.279
Fibrinogen—mg/dL, median (IQR)	392.00 (311.00–491.00)	494.00 (381.50–544.25)	*U* = 883, *Z* = −2.548	0.011
Hemoglobin—g/dL, median (IQR)	9.80 (8.60–10.85)	10.50 (9.20–11.95)	*U* = 1179.5, *Z* = −1.836	0.066
Thrombocytes × 10^3^/µL, median (IQR)	214.50 (136.75–296.50)	225.00 (168.00–295.50)	*U* = 1350, *Z* = −0.811	0.417
Ultrasensitive Troponin I—ng/mL, median (IQR)	32.50 (17.50–49.12)	29.00 (6.20–36.70)	*U* = 511, *Z* = 1.068	0.286
D-dimers—ng/mL, median (IQR)	2.50 (1.55–4.05)	1.47 (0.60–2.23)	*U* = 671, *Z* = 2.734	0.006
Creatinine—mg/dL, median (IQR)	1.00 (0.80–1.69)	1.00 (0.78–1.77)	*U* = 1540.5, *Z* = 0.334	0.739
GOT—U/L, median (IQR)	44.00 (30.25–60.50)	24.00 (16.75–84.00)	*U* = 1147.5, *Z* = 1.538	0.124
LDH—U/L, median (IQR)	308.00 (213.00–388.00)	222.50 (166.25–298.75)	*U* = 961, *Z* = 2.866	0.004
Total bilirubin—mg/dL, median (IQR)	0.87 (0.7–1.1)	0.8 (0.43–0.90)	*U* = 603, *Z* = 1.665	0.096

**Table 7:** Complications and clinical outcomes—comparison between the two groups.

	*Staphylococcus* spp. IE (*n* = 66)	Non-*Staphylococcus* spp. IE (*n* = 46)	Test Statistic Value	*p*-Value
Any embolic event, *n* (%)	36 (54.5%)	14 (30.4%)	*ꭓ*^2^ = 6.376	0.012 ^a^
Pulmonary embolism, *n* (%)	23 (34.8%)	4 (8.7%)	-	0.001 ^b^
Hepato-splenic embolism, *n* (%)	4 (6.1%)	5 (10.9%)	-	0.483 ^b^
Cerebral/meningeal embolism, *n* (%)	13 (19.7%)	6 (13.0%)	*ꭓ*^2^ = 0.852	0.356 ^a^
ICU admission, *n* (%)	17 (25.8%)	6 (13.0%)	*ꭓ*^2^ = 2.685	0.101 ^a^
Cardiac surgery consult, *n* (%)	14 (21.2%)	10 (21.7%)	*ꭓ*^2^ = 0.004	0.947 ^a^
Cardiac surgery performed, *n* (%)	7 (10.6%)	8 (17.4%)	*ꭓ*^2^ = 1.076	0.300 ^a^
Discharged to other facility, *n* (%)	5 (7.6%)	1 (2.2%)	-	0.398 ^b^
Discharge improved, *n* (%)	46 (69.7%)	33 (71.7%)	*ꭓ*^2^ = 0.054	0.816 ^a^
Hospital stay—days, median (IQR)	31.5 (14.8–44.3)	30.0 (14.8–42)	*U* = 1615.4, *Z* = 0.571	0.568
In-hospital mortality, *n* (%)	12 (18.2%)	4 (8.7%)	*ꭓ*^2^ = 1.992	0.158 ^a^

^a^—Chi-squared test, ^b^—Fisher’s exact test.

**Table 8:** Multivariable predictors of Staphylococcal etiology.

	Odds Ratio (OR)	95% CI Lower Limit	95% CI Upper Limit	*p*-Value
IVDU	7.099	2.446	20.605	<0.001
CRP at admission	1.077	1.009	1.148	0.025
SaO_2_ (%)	0.747	0.604	0.924	0.007

IVDU—intravenous drug use; CI—confidence interval.

**Table 9:** Simplified bedside risk score for predicting Staphylococcal IE.

Variable	β-Coefficient	Scaling	Point Assignment	Rationale
Intravenous drug use (IVDU)	1.96	binary	+5 points	Strongest independent predictor (highest regression coefficient)
C-reactive protein (CRP)	0.74	per 10 mg/dL increase	+2 points	Reflects inflammatory burden
Oxygen saturation (SaO_2_)	0.29	per 1% decrease	+1 point	Reflects pulmonary involvement (e.g., septic emboli)

**Table 10:** Distribution of *Staphylococcus* IE probability and clinical interpretation by risk group.

Score Group	Score Range	Non-Staphylococcal IE	Staphylococcal IE	Observed Risk	Clinical Interpretation
Low	<7	37 (72.5%)	14 (27.5%)	28%	*Staphylococcus* less likely
Intermediate	7–11	5 (17.9%)	23 (82.1%)	82%	strong suspicion
High	>11	2 (6.5%)	29 (93.5%)	94%	very high probability

**Table 11:** Differences in Platelet Count Changes and Recovery According to IE Etiology.

Variable	Non-Staphylococcal IE (*n* = 46)	Staphylococcal IE (*n* = 66)	*U* Statistic	*p*-Value
Δ Platelet count (cells/µL), median (IQR)	−35,000 (141,000)	43,000 (143,250)	1037.5	0.007
Platelet recovery, median (IQR)	−0.102 (0.562)	0.246 (1.57)	1000.5	0.004

Δ Platelet count—the absolute change in platelet count between hospital discharge and admission. Platelet recovery—the proportional change in platelet count during hospitalization. Continuous variables are presented as median (IQR) and compared using the Mann–Whitney *U* test.

## Data Availability

The data presented in this study are available on request from the corresponding author.

## Supplementary Materials

The following supporting information can be downloaded at: https://www.mdpi.com/article/10.3390/pathogens15040418/s1, Table S1: Species distribution in patients with Staphylococcal Infective Endocarditis (IE), Table S2: Species distribution in patients with Non-Staphylococcal Infective Endocarditis (IE).

## Abbreviations

The following abbreviations are used in this manuscript:

AUC	Area Under the Curve
AV	Aortic Valve
BMI	Body Mass Index
CCI	Charlson Comorbidity Index
CI	Confidence Interval
CoNS	Coagulase-negative staphylococci
CRP	C-Reactive Protein
DCA	Decision Curve Analysis
GOT	Glutamic Oxaloacetic Transaminase (Aspartate Aminotransferase, AST)
HIV	Human Immunodeficiency Virus
HCV	Hepatitis C Virus
HBV	Hepatitis B Virus
IE	Infective Endocarditis
IQR	Interquartile Range
IVDU	Intravenous Drug Use
LDH	Lactate Dehydrogenase
MV	Mitral Valve
OR	Odds Ratio
ROC	Receiver Operating Characteristic
SaO_2_	Peripheral Oxygen Saturation
SD	Standard Deviation
SOFA	Sequential Organ Failure Assessment
spp.	Species (plural, used after genus name)
TRIPOD	Transparent Reporting of a Multivariable Prediction Model for Individual Prognosis or Diagnosis
TTE	Transthoracic Echocardiography
TEE	Transesophageal Echocardiography
TV	Tricuspid Valve
PV	Pulmonary Valve
Δ	Delta (change or variation)
