# Supplementary material for: Phenotype-Based Stratification and Early Prediction of Staphylococcal Infective Endocarditis: Development of a Pragmatic Bedside Score
Source: Pathogens. 2026 Apr 13;15(4):418. doi: 10.3390/pathogens15040418 (PMC13118454; doi:10.3390/pathogens15040418)
Supplement: Supplementary file 1 [file pathogens-15-00418-s001.zip › pathogens-4242092-supplementary.pdf]

Table S1. Species distribution in patients with Staphylococcal Infective Endocarditis (IE)

Species identified in the Staphylococcal IE group	Frequency	Percent (%)
MSSA	30	45.5
MRSA	25	37.8
Staphylococcus epidermidis	4	6.1
Staphylococcus hominis	3	4.5
Coagulase-negative *Staphylococcus*, no further specified	2	3.0
Staphylococcus capitis	1	1.5
Staphylococcus lugdunensis	1	1.5
Total	66	100.0

Table S2. Species distribution in patients with Non-Staphylococcal Infective Endocarditis (IE)

Species identified in the Non-Staphylococcal IE group	Frequency	Percent (%)
Coxiella burnetii	4	8.7
Enterococcus faecalis	9	19.6
Enterobacter aerogenes	1	2.2
Enterobacter cloacae	1	2.2
Escherichia coli	1	2.2
Klebsiella pneumoniae	2	4.3
Proteus mirabilis	1	2.2
Pseudomonas aeruginosa	1	2.2
Streptococcus pneumoniae	1	2.2
Streptococcus agalactiae	1	2.2
Streptococcus anginosus	1	2.2
Streptococcus constellatus	1	2.2
Streptococcus dysgalactiae	1	2.2
Streptococcus gallolyticus	8	17.4
Streptococcus gordonii	2	4.3
Streptococcus mitis	2	4.3
Streptococcus oralis	3	6.5
Streptococcus pyogenes	1	2.2
Streptococcus salivarius	1	2.2
Streptococcus sanguinis	1	2.2
Streptococcus viridans	2	4.3
**Total**	46	100
